# Development and validation of a flax (*Linum usitatissimum *L.) gene expression oligo microarray

**DOI:** 10.1186/1471-2164-11-592

**Published:** 2010-10-21

**Authors:** Stéphane Fenart, Yves-Placide Assoumou Ndong, Jorge Duarte, Nathalie Rivière, Jeroen Wilmer, Olivier van Wuytswinkel, Anca Lucau, Emmanuelle Cariou, Godfrey Neutelings, Laurent Gutierrez, Brigitte Chabbert, Xavier Guillot, Reynald Tavernier, Simon Hawkins, Brigitte Thomasset

**Affiliations:** 1 Université Lille Nord de France, Lille 1 UMR INRA 1281, SADV, F- 59650 Villeneuve d'Ascq cedex, France; 2 EA 3900-BioPI, UFR des Sciences, UPJV, 33 rue Saint Leu, 80039 Amiens cedex, France; 3 BIOGEMMA, Z.I. du Brezet, 8 rue des Frères Lumières, 63028 Clermont-Ferrand cedex 2, France; 4 BIOGEMMA, domaine de Sandreau, Chemin de Panedautes, 31700 Mondonville, France; 5 Institut Technique de Lin, 27170 Ecardenville La Campagne, France; 6 CRRBM, UFR des Sciences, UPJV, 33 rue Saint Leu, 80039 Amiens cedex, France; 7 UMR- INRA, UMR614, URCA, FARE, 2 Esplanade R. Garros, CREA, BP 224, 51686 Reims, France; 8 Laboulet Semences, 80 270 Airaines, France; 9 LINEA, 20 Avenue Saget, 60 210 Grandvilliers, France; 10 UMR CNRS 6022, GEC, Université de Technologie de Compiègne, BP 20529, 60205 Compiègne cedex, France

## Abstract

**Background:**

Flax (*Linum usitatissimum *L.) has been cultivated for around 9,000 years and is therefore one of the oldest cultivated species. Today, flax is still grown for its oil (oil-flax or linseed cultivars) and its cellulose-rich fibres (fibre-flax cultivars) used for high-value linen garments and composite materials. Despite the wide industrial use of flax-derived products, and our actual understanding of the regulation of both wood fibre production and oil biosynthesis more information must be acquired in both domains. Recent advances in genomics are now providing opportunities to improve our fundamental knowledge of these complex processes. In this paper we report the development and validation of a high-density oligo microarray platform dedicated to gene expression analyses in flax.

**Results:**

Nine different RNA samples obtained from flax inner- and outer-stems, seeds, leaves and roots were used to generate a collection of 1,066,481 ESTs by massive parallel pyrosequencing. Sequences were assembled into 59,626 unigenes and 48,021 sequences were selected for oligo design and high-density microarray (Nimblegen 385K) fabrication with eight, non-overlapping 25-mers oligos per unigene. 18 independent experiments were used to evaluate the hybridization quality, precision, specificity and accuracy and all results confirmed the high technical quality of our microarray platform. Cross-validation of microarray data was carried out using quantitative qRT-PCR. Nine target genes were selected on the basis of microarray results and reflected the whole range of fold change (both up-regulated and down-regulated genes in different samples). A statistically significant positive correlation was obtained comparing expression levels for each target gene across all biological replicates both in qRT-PCR and microarray results. Further experiments illustrated the capacity of our arrays to detect differential gene expression in a variety of flax tissues as well as between two contrasted flax varieties.

**Conclusion:**

All results suggest that our high-density flax oligo-microarray platform can be used as a very sensitive tool for analyzing gene expression in a large variety of tissues as well as in different cultivars. Moreover, this highly reliable platform can also be used for the quantification of mRNA transcriptional profiling in different flax tissues.

## Background

Flax (*Linum usitatissimum *L.) has long been cultivated by man for its cellulose-rich bast fibres and seeds [1,2]. The long bast fibres are traditionally used in the textile industry for the production of linen or mixed fibre textiles, and, together with shorter xylem fibres are also used in the automobile and construction industries [3,4]. Flax seeds are widely integrated into animal feeds [5] and are also important in human health since they are a major source of omega-3 fatty acids [6] and biologically active lignan [7]. Linseed oil from seeds is equally used in the fabrication of paint, lacquer and varnish, soap, putty and polymers [8,9].

In order to breed improved flax varieties we need to increase our fundamental knowledge of flax biology (fibre and seed formation, disease resistance, growth etc.) since many important questions remain unanswered. For example, although we know that fibre quality is related to the extremely low lignin levels found in this cell type as compared to wood fibres, we do not know how lignin biosynthesis is regulated in flax fibres [10]. Similarly, although the different biosynthetic pathways involved in fatty acid production in linseed are known, we still know relatively little about the biological mechanisms controlling linolenic acid levels in traditional flax varieties (45-65% omega-3 fatty acid) compared to solin or linola varieties (2%) [11,12].

The size of the flax genome (686 Mbp) is around four times that of Arabidopsis and recently different research teams have developed reverse genetics and genomic approaches to learn more about fibre and seed formation in this economicly-important species [13-18]. A cDNA custom flax array system has recently been constructed and successfully used to characterize genes expression profiles in fibre-bearing stem tissues [17,18]. This platform was constructed by spotting 9,600 anonymous cDNA clones obtained from a flax stem-peel cDNA library. In this paper we report the development and validation of a flax-specific high-density oligo-microarray platform using Nimblegen technology. Flax cDNAs generated from different tissues and/or developmental stages from two different flax cultivars (oil-seed and fibre flax respectively) were 454-sequenced and assembled into contigs. These contigs representing genes found in both oil-seed and fibre flax were used to produce a 48K array. The high quality of our array was demonstrated by the high reproducibility of different technical replications, as well as by the platform's capacity to identify differential gene expression profiles in different tissues and flax varieties.

## Results and discussion

### 454 Sequencing and contig assembly

In order to produce representative high-density flax (*Linum usitatissinum*) microarrays, we extracted RNA from 9 different samples (Table 1) corresponding to different tissues and/or developmental stages from two flax cultivars (Barbara, an oil-seed cultivar, and Hermes, a fibre cultivar) and prepared them for 454 sequencing. Different samples were chosen for their biological interest e.g. young- (10-15 Days After Flowering, DAF), mid-stage- (20-30 DAF) and maturing- (40-50 DAF) seeds correspond to early, maximal and late stages of storage compounds synthesis. Similarly, vegetative growth (50-60 days after germination, DAG) and green capsule stages (70-80 DAG) are associated with fibre development.

**Table 1:** Details of flax samples used for GS FLX sequencing and for microarray validation experiments.

Roots (Hermes)	Leaves (Hermes)	Stems (Hermes)		Seeds (Barbara)
(**R**)	(**L**)	Outer tissue	Inner tissue	10-15 DAF (**S1**)
		
		Vegetative phase (**SOV**)	Vegetative phase (**SIV**)	20-30 DAF (**S2**)
		
		Green capsule phase (**SOGC**)	Green capsule phase (**SIGC**)	40-50 DAF (**S3**)

R = roots; L = leaves; SOV = stem outer tissues; vegetative stage; SIV = stem inner tissues; vegetative stage; SOGC = stem outer tissues, green capsule stage; SIGC = stem inner tissues, green capsule stage; S1 = seeds, 10-15 Days After Flowering (DAF); S2 = seeds, 20-30 DAF, S3 = seeds, 40-50 DAF.

Four 454 GSFLX half-runs were used to sequence the 9 samples generating 1,068,375 reads with an average length of 281 bp and a total of 287 Mb (Table 2). MIDs (Multiplex identifiers) were used to mix samples into the same half-runs: SOV/SOGC, SIV/SIGC, L/R, and S1/S2/S3. Individual sample data was then separated from the four SFF (Standard Flowgram Format) files using the Roche 454 SFF info tool.

**Table 2:** 454 GS FLX sequencing data for the 9 samples

Tissues/Samples	Reads #	Average length	Bases #	Bases after clip #
SOV	162,256	289.62	43,779,333	33,153,393

SOGC	112,872	275.58	30,082,124	23,827,193

SIV	133,816	294.61	35,475,882	27,206,866

SIGC	154,790	299.80	41,108,455	31,266,452

L	134,342	286.15	36,243,207	26,340,221

R	131,051	273.08	35,614,353	24,190,625

S1	76,232	259.95	20,913,548	14,693,725

S2	80,148	252.54	22,077,809	14,419,155

S3	82,868	272.14	22,203,052	16,630,053

**All**	**1,068,375**	**281.67**	**287,497,763**	**211,727,683**

After cleaning 881,950 reads were assembled into 59,494 contigs, with 132 singlets giving a total of 59,626 unigenes http://urgi.versailles.inra.fr/index.php/urgi/Species/Flax/Dowload-sequences. The N50 was 569 bp and 17,487 contigs were longer than 500 bp. 17,731 contigs had only 2 reads, >15,000 contigs contained more than 10 reads and >8,500 contigs contained more than 20 reads. The number of reads varied from 1 to more than 3,500 potentially reflecting the relative abundance of the different transcripts. Incorporation of Sanger sequences available in the public domain did not alter our assembly suggesting that the shorter reads generated by 454 sequencing did not represent a problem for correct assembly (data not shown).

### Functional Characterization, GO notation

**Figure 1:**
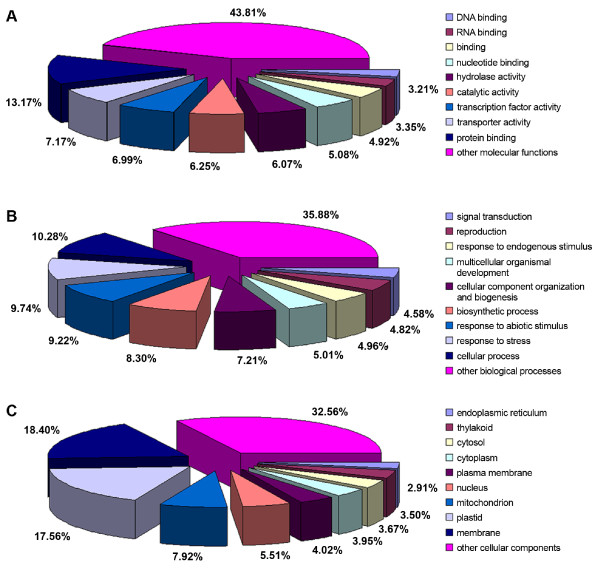
**Summary of predicted gene product function and location using gene ontology terms**. The data was obtained from the set of flax unigenes derived from the assembly of the 454 sequences. A: Molecular function; B: Biological process; C: Cellular component.

Contigs were loaded into the EST2uni database and the corresponding pipeline was used to make all annotations. BLAST analyses were used to compare flax contig sequences to 4 different databases (TAIRV7_cds, TAIRV7_pep, EMBL_plant and UNIPROT_plant; Table 3). Between 21.3% and 62.9% contigs show significant similarity to known genes (evalue ≤ e-20), depending upon the database used for comparison and putative Arabidopsis orthologs were found for 14.7% flax contigs. The relatively low value is probably to be expected since the reciprocal blasts necessary to identify putative orthologs was performed between the non-redundant Arabidopsis gene data set and the 59K flax unigene set. GO annotations could be assigned to approximately 25% of the unigenes by similarity using blast results and GOA and TAIR gene cross-referenced files. 10,010 sequences (16.8%) could be assigned molecular functions (Figure 1A), 14,501 sequences (24.3%) could be assigned biological processes (Figure 1B) and 16,570 sequences (27.8%) sequences could be assigned cellular components (Figure 1C).

**Table 3:** Numbers and percentages of flax unigenes showing blast hits against different databases

Database	Number of flax unigene hits	%
TAIRV7_cds	12672	21.3

TAIRV7_pep	32399	54.3

EMBL_plant	22804	38.2

UNIPROT_plant	34473	57.8

Any database	37490	62.9

### Microarray Design

Analyses of the 59K unigene set allowed for several different probe design possibilities. After examination, we chose a design based upon 48K contigs as being the most representative of the 59K unigene set. Microarrays were designed according to the Roche/Nimblegen protocol based on 3' end cDNA synthesis and both annotated and non-annotated contigs were used. The final design contained 8 non-overlapping oligos (25-30 mers) per contig allowing for both qualitative and quantitative hybridization. This design is available at the GEO web site http://www.ncbi.nlm.nih.gov/projects/geo/ under the platform number GPL104.

### Parameters of microarray performance

The Nimblegen array system is based upon the hybridization of a single labeled sample (derived from RNA), followed by one-channel detection. The intensity of the hybridization signal is then used to determine target concentration. In order to check the technical quality of each probe in our flax Nimblegen arrays, we performed 18 independent hybridizations on nine samples representing different tissues and/or different developmental stages (Table 1) with two technical repetitions. In order to avoid any potential bias due to biological variability the hybridizations were performed on the same RNA samples (Table 1) used for 454 sequencing. Technical quality was determined by evaluating the following parameters: hybridization quality, precision and accuracy.

#### Hybridization quality

Our results (GEO GSE21868) showed that all probes present on the array were capable of hybridizing successfully (signal > background) when tested on the different samples. The sensitivity of the array was demonstrated by the wide signal dynamic range obtained (log2 values of 4 to 16). The experimental metrics report (NimbleScan v2.5) was used as recommended by Roche/Nimblegen to generate summary statistics that can be used to identify any potential problems during hybridization. Most of these metrics (interquartile density, signal range, uniformity mean, uniformity CV (coefficient of variation), number of empty features on the array, mean empty, the number of random control features present on the array, mean Random) assume probe randomization on the array surface. Thus, deviation from uniformity (i.e. outside the recommended value range) across the array could suggest potential artifacts during hybridization. Metric values (data not shown) for all 18 samples analyzed were within the recommended value range indicating that hybridization quality was satisfactory.

#### Precision

**Figure 2:**
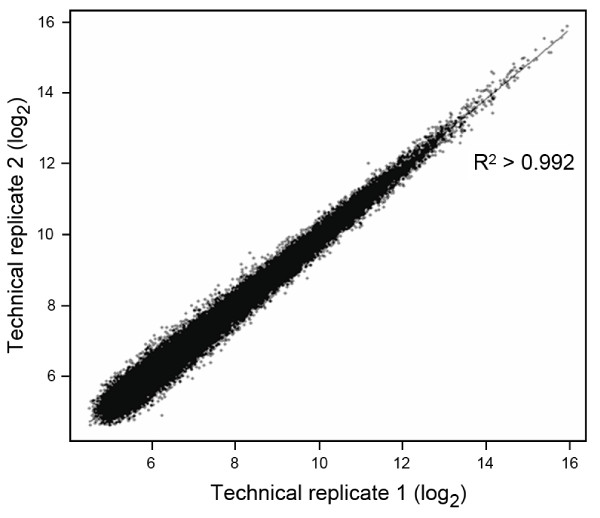
**Microarray reproducibility**. Scatter plot showing correlation between 2 microarray technical replicates (example shown on flax leaf sample, L). High correlations were found between all technical replicates (r = 0.98 or higher, p-value < 2.2e^-16^).

A crucial aspect of all microarray experiments is good system reproducibility enabling direct and reliable comparisons between different experiments. Precision describes how accurately the hybridization signal intensity can be reproduced and is usually reported as a correlation coefficient, standard deviation or average replicate error between duplicated experiments using the same RNA sample [19]. Raw expression data on the 18 (9 × 2) flax hybridization experiments were normalized and Pearson's correlation coefficients were calculated for the data sets of hybridization signal intensities. High correlation coefficients were obtained in all cases and the results obtained for the flax leaf sample are shown in Figure 2. These results (r ≥ 0.98; p-value < 2.2e^-16 ^) indicate an almost perfect correlation between different technical experiments underlining the extremely high precision level for our flax oligo-array platform. Such reproducibility is similar to that obtained with Affymetrix GeneChips [20] and is probably also due to probe redundancy. Previous work has shown that the use of multiple independent oligonucleotides designed to hybridize to different regions of the same RNA improves signal-to-noise ratio and the dynamic range of detection, as well as minimizing cross-hybridization effects [21].

#### Accuracy

Accuracy describes how close to a true value a measurement lies. It can be estimated in experiments where a number of realistic targets are spiked at known concentrations into relevant RNA populations, or from comparisons with validation experiments, or by correlation of two different methods [19].

**Figure 3:**
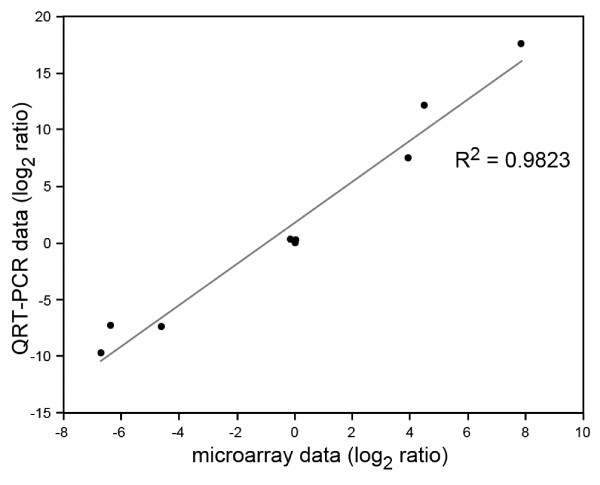
**Correlation between qRT-PCR and microarray results**. A statistically significant correlation (r = 0.9823, p = 2.376e^-06^) was obtained for all tested genes.

Gene-specific quantitative qRT-PCR was used for the cross-validation of platform performance and as an assessment of microarray accuracy. We selected 9 genes reflecting up-regulated (c20715, c2491 c602), down-regulated (c2533, c3323, c4370) or equally-expressed genes (c21991, c24118, c9380) in leaf (L) samples compared with stem (SIGC) samples. Selected genes also covered a wide range of signal intensity (5.3849-14.0473). Table 4 shows the expression levels of the same target genes detected by qRT-PCR and microarray analyses. Expression level was calculated as log2ratio of mean signal intensity across three technical replicates between L and SIGC samples. A statistically significant correlation (r = 0.9823, p = 2.376e^-06^) between qRT-PCR and microarray results was obtained for all tested genes (Figure 3) indicating high concordance between these 2 data sets. The observation that the fold-change values in gene expression are lower for the microarray data as compared to the qRT-PCR data is most likely due to data compression resulting from limited dynamic range or signal saturation [22]. These data indicate that our oligo-array platform is able to accurately predict the direction of change of gene expression level (i.e. either up or down regulation) between subsets of interest.

**Table 4:** Comparison of expression levels (log2ratio) from qRT-PCR and microarray for selected target genes

Target Transcript	Gene ID	Microarray value (Log2ratio)	qRT-PCR value (Log2ratio)
Lipoxygenase LOX2	c20715	7.86465	17.6

Chlorophyll a-b binding protein 3C-like	c2491	3.9446	7.47

RuBisCO activase 2	c602	4.5263	12.185

DNA-directed RNA polymerase II	c21991	0.01865	-0.015

Calmodulin TaCaM2-1	c24118	-0.10225	0.295

Ubiquitin carboxyl- terminal hydrolase	c9380	0.05875	0.215

Cellulose synthase	c2533	-4.5911	-7.37

Laccase	c3323	-6.3722	-7.31

Fasciclin-like AGP 4	c2370	-6.7035	-9.72

Gene expression was calculated as log2ratio of L *vs *SIGC samples, using mean signal intensity across three technical replicates of each sample. Values >1 indicate up-regulation, and values <-1 indicate down-regulation.

### Differential gene expression detection

In order to see whether our flax microarray platform was able to generate biologically-useful information, we analyzed its capacity to detect specific gene expression profiles associated with different tissues, different developmental stages, and different genotypes. Firstly, we used principal component analysis (PCA) to compare expression profiles in the 9 different samples previously hybridized. Secondly, we compared the expression of 4 genes known to be involved in the process of secondary cell wall biosynthesis and wood formation. Thirdly, we directly compared expression profiles between inner and outer stem tissues at two developmental stages. Finally, we compared gene expression profiles in 2 supplementary flax varieties that show differences in fibre quality and disease resistance.

#### Differential gene expression in flax tissues and developmental stages

**Figure 4:**
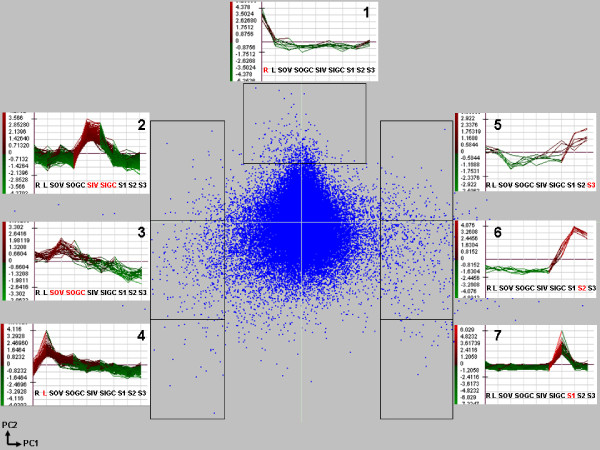
**Principal component analysis (PCA) of microarray data and representative K-means profiles of tissue-specific gene expressions**. Variance of principal component 1 (PC1) = 41.05% and of principal component 2 (PC2) = 17.73%. Three major regions of dispersion were found representing A) root specific genes (profile 1); B) stem specific genes (profiles 2 and 3) and leaf specific genes (profile 4); and C) seed specific genes (profiles 5, 6 and 7). Genes similarly expressed in different tissues are located in the middle of the PCA figure. R: root; L: leaf; SOV: Shoot outer tissue, vegetative stage; SOGC: shoot outer tissue, green capsule stage; SIV: Shoot inner tissue, vegetative stage; SIGC: shoot inner tissue, green capsule stage; S1: 10-15 days-old seeds; S2: 20-30 days-old seeds; S3: 40-50 days-old seeds.

The results of principal component analysis (PCA) of microarray data and representative K-means profiles of tissue-/stage-specific gene expressions are shown in Figure 4. Three major regions of dispersion are found: A) genes specifically expressed in roots (Figure 4, top, profile 1), B) genes expressed specifically in stems and leaves (Figure 4, left, profiles 2-4) and C) genes specifically expressed in seeds (Figure 4, right, profiles 5-7). Genes similarly expressed in all tissues are clustered in the middle of the figure.

**Figure 5:**
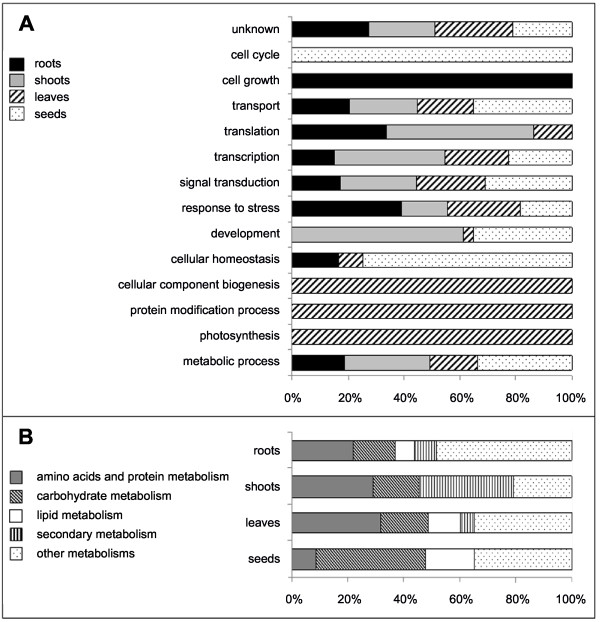
**GO unigene annotation on microarray data**. A - Percentage representation of differentially expressed genes from different flax tissues in different GO biological processes. B - Percentage representation of differentially expressed genes from different flax tissues in different metabolisms.

J-Express generated a list of 1,357 specifically expressed genes of which 609 were root-specific genes, 599 leaf-specific genes, 79 shoot-specific genes and 70 seed-specific genes. Differential expression profiles of these genes were verified by a SAM test with a FDR less than 5%. Annotated genes in these different groups were then classified into different GO biological processes and the percentages of tissue-specific gene expressions were calculated for each process (Figure 5A).

Overall, these results are in general agreement with the known physiological processes of the different organs/tissues thereby suggesting that our platform is capable of generating biologically-useful gene-expression data. For example, leaves are well known to be the plant's main photosynthetic organs and leaf structure is closely associated with its photosynthetic function and chloroplast biogenesis [23-31]. The observation that the GO process 'photosynthesis' is entirely composed of genes specifically expressed in the leaves (Figure 5A), as are the GO processes 'cellular component biogenesis', and 'protein modification process' is in agreement with the biological structure and role of this organ. In root tissues the GO processes 'cell growth' and 'response to stress' were the most represented functions in agreement with the biological activities of this organ [32,33]. As in other plant species, the flax stem is characterized by the differentiation of different specialized tissues [18,34] and the majority stem-specific gene expression in the development GO process is in keeping with the biological activity of this organ. Finally, cell-cycle and cellular homeostasis GO processes were associated with embryo-specific gene expressions characteristic of embryogenesis [35].

Similar analyses (Figure 5B) of the relationship between different tissue-specific genes and metabolisms also suggest that our platform is capable of generating biologically-relevant information. Of interest are the relatively high percentages of genes associated with lipid and carbohydrate metabolisms in seeds since direct quantitative measurements [36] have shown that sucrose and lipids represent the largest metabolite pools in flax seeds. Similarly, secondary metabolism represents the major metabolism in flax stems and is most likely associated with the lignification of xylem secondary cell walls [34].

#### Differential gene expression in a targeted process

**Figure 6:**
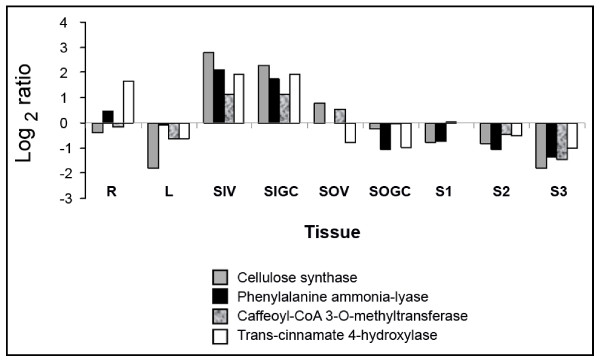
**Expression levels of 4 genes involved in secondary cell wall biosynthesis and wood formation**. Gene expression was calculated as log2ratio of the signal intensity in each tissue *vs*. The average of signal intensity across all analyzed tissues.

We compared the expression of four genes known to be involved in the process of secondary cell wall biosynthesis and wood formation characteristic of inner stem tissues [37]. Expression levels of *cellulose synthase *(c2533), *phenylalanine ammonia-lyase *(c247), *transcinnamate 4-hydroxylase *(c11079), and *caffeoyl-CoA 3-O-methyltransferase *(c15771) were calculated as log2ratio of the signal intensity in each tissue *vs*. the average of signal intensity across all analyzed tissues (Figure 6). As expected, the great majority of these genes were more highly expressed in inner tissues (at both developmental stages examined). These observations confirm that the flax oligo-array platform is able to accurately discriminate gene expression profiles in different tissues.

#### Differential gene expression between flax inner and outer stem tissues

In order to verify that our oligo-array platform could be used in future studies to provide biologically-relevant information about cell wall formation and fibre development in flax, we compared expression profiles between the heavily lignified inner stem tissues and the outer stem tissues enriched in weakly-lignified bast fibres at two different developmental stages.

The results (Additional files 1 and 2) showed that in vegetative stage samples, 203 genes were over-expressed in inner stem tissues as compared to outer stem tissues (cut-off: log2ratio (SIV/SOV) > 2), and 229 genes were over-expressed in outer stem tissues as compared to inner stem tissues. Examination of the 203 genes over-expressed in the inner stem tissues showed that 56 genes (28%) were potentially associated with cell wall formation and xylem identity. Of these differentially-expressed cell wall genes, 50% (28 genes) were associated with lignification and included both monolignol biosynthetic genes and laccases involved in the oxidative polymerisation of this phenolic polymer. Such an observation is in agreement with the fact that flax inner stem tissues, mainly composed of xylem, are much more heavily lignified than the outer tissues containing cellulose-rich bast fibes [34]. Other cell-wall related genes coded for proteins involved in polysaccharide cell wall polymer synthesis and remodelling. In addition, 3 genes (C1109, C3396, C5556) coding for fasciclin-like arabinogalactan proteins (FLAs) were also over-expressed in inner tissues as compared to outer tissues. Interestingly, FLA genes are highly expressed in flax *outer *stem tissues, as well as in poplar tension wood where they have been hypothesized to be involved in the formation of cellulose-rich gelatinous fibres (g-fibres) [17,37,38]. Our observation (see below) that 3 other FLA genes (C2947, C3576, C5237) are more highly expressed in flax outer stem tissues when compared to inner stem tissues could suggest that these proteins play a role in secondary cell wall formation in both flax inner and outer stem tissues.

Similar analyses (Additional files 1 and 2) of the 229 genes over-expressed in flax outer stem tissues when compared to inner stem tissues revealed that only 16 genes (7%) were associated with cell wall formation. In contrast, 25 genes were potentially associated with lipid and wax metabolism, 8 genes were related to photosynthesis and 8 genes were stress-related. These differences reflect the different physiological status of flax inner and outer stem tissues. For example, the high percentage of 'cell-wall-related' transcripts in inner stem tissues is associated with secondary xylem formation while the relative abundance of photosynthesis-related transcripts reflects the fact that outer stem tissues are green. Nevertheless, it is interesting to note that 6 genes (C1423, C6325, C353, C1410, C8886, C5052) over-expressed in outer stem tissues corresponded to Lipid Transfer Proteins (LTPs). LTPs have been previously associated with diverse aspects of cell wall development and formation and have also been shown to be more highly expressed in flax outer stem tissues [17,39]. Such an observation could suggest that such proteins might be associated with fibre maturation in flax. As indicated above, 3 FLAs (C2947, C3576, C5237) were also over-expressed in outer stem tissues as compared to inner stem tissues.

Comparison (Additional files 1 and 2) of inner and outer stem tissue expression profiles (cell-wall related genes) at the green capsule stage revealed a very similar differential expression pattern to that observed for the vegetative stage suggesting that little modification/evolution of inner and outer stem transcriptomes occurs between these two developmental stages. This was confirmed by the observation that only 3 cell-wall related genes (C25634, C2733, C11945) were up-regulated in inner stem tissues at the vegetative stage as compared to the green capsule stage, and no cell-wall related genes were up-regulated in inner stem tissues at the green capsule stage as compared to vegetative inner stem tissues. In outer stem tissues, 8 cell wall genes (C31544, C37539, C6312, C59350, C6820, C44241, C5939, C51183) were more highly expressed at the vegetative stage as compared to the green capsule stage. Two of these genes (C44241, C5939) code for xyloglucan endotransglycosylase/hydroalses (XTHs), one gene (C6820) codes for a secondary cell wall associated glycosyltransferase and one gene for a FLA. Since these 4 genes are implicated in cell wall remodelling and assembly events, such an observation could suggest that cell wall formation in outer stem tissues is more active at the vegetative stage as compared to the green capsule stage. No cell wall genes were more highly expressed at the green capsule stage as compared to the vegetative stage.

The vegetative stage (7-8 weeks) corresponds to the fast-growth stage associated with the increase in plant height and fibre lengthening above the snap point, as well as fibre cell wall thickening below the snap point [40]. At the green capsule stage (10-11 weeks) fibres have stopped elongating and further fibre development is restricted to continued fibre cell wall thickening [40,41]. Our results would suggest that continued fibre thickening (as well as continued secondary xylem formation in flax inner stem tissues) is associated with continued expression of cell wall related genes.

Altogether these analyses confirm that our oligo-array platform represents a powerful tool for investigating cell wall development and fibre formation in flax stems. Our arrays should also prove extremely useful for investigating other interesting biological processes in flax such as oil and seed formation, disease resistance and tolerance to abiotic stress.

#### Differential gene expression in contrasting flax genotypes

We used our platform to see whether the flax microarrays were able to detect differentially-expressed genes between 2 contrasting flax genotypes - Drakkar and Belinka. Drakkar produces better quality fibres than the variety Belinka, as well as being more resistant to the fungal pathogen *Fusarium *http://www.lin-itl.com[42]. RNA was isolated at the green capsule stage from outer stem tissues of field-grown flax plants and 3 biological and 2 technical repetitions were used for each variety. Following hybridization, the Log2ratio was calculated as the intensity signal of each gene in the Drakkar genotype *vs*. Belinka. The Pearson's correlation was > 0.99 for technical repetitions and > 0.97 for biological repetitions for both Belinka and Drakkar. A total of 428 up-regulated genes and 367 down-regulated genes were found. Only 269 genes were annotated or associated with a known biological function. (Additional files 3 and 4).

**Figure 7:**
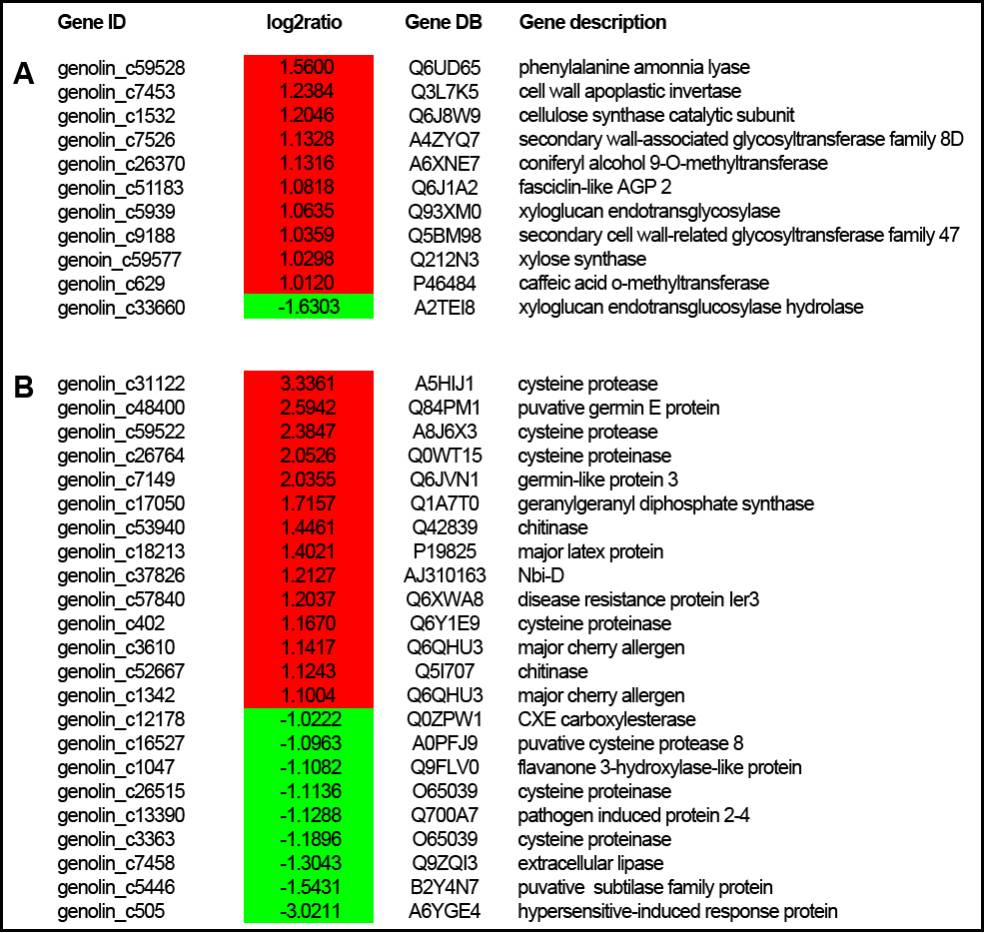
**Differentially expressed genes in two cultivars, Drakkar and Belinka**. Differences concern genes involved in cell wall biosynthesis (A) and response to biotic stimuli (B). Log2ratio were calculated as intensity signal in Drakkar *vs*. Belinka. Values for up-regulated genes are represented in red and those for down-regulated genes in green colour.

Since the 2 genotypes show differences in fibre quality and pathogen resistance, we focused our attention on those differentially-expressed genes associated with cell wall biosynthesis and response to biotic stimuli. Fibre quality in flax is associated with both the structure of the cellulose-rich secondary cell wall and the architecture (length, diameter) of individual fibres [16,34,43]. The observation (Figure 7A) that 10 cell-wall related genes were up-regulated in Drakkar, as compared to Belinka is therefore extremely interesting. These genes include 1 gene (*cellulose synthase catalytic subunit *- c1532) involved in cellulose biosynthesis, 3 genes (*xylose synthase *- c59577; *secondary cell wall-related glycosyltransferase family 47 *- c9188; *secondary wall-associated glycosyltransferase family 8 D *- c7526) involved in secondary cell wall hemicellulose synthesis, 3 genes (*phenylalanine amonnia lyase *- c59528; *caffeic acid o-methyltransferase *- c629; *coniferyl alcohol 9-O-methyltransferase *(c26370), involved in phenylpropanoid/lignin biosynthesis, 1 gene (*xyloglucan endo-transglycosylase *- c5939) involved in cell wall expansion, 1 gene (*cell wall apoplastic invertase *- c7453) involved in sucrose partitioning [44], and 1 gene (*fasciclin-like AGP 2 *- c51183) that has previously been associated with both flax fibre formation [45] and G-fibre formation in tree reaction wood [46,47]. Only 1 cell wall related gene (*xyloglucan endotransglucosylase hydrolase *- c33660) associated with wall remodeling events was up-regulated in Belinka as compared with Drakkar. The up-regulation of these genes could suggest that cell wall biosynthesis is more dynamic in the Drakkar variety and is possibly related to the different fibre quality characteristic of this variety. However, further functional validation is obviously necessary before confirming such hypotheses.

A total of 14 genes involved in biotic stress response were up-regulated in Drakkar as compared with Belinka, while 9 genes were up-regulated in Belinka as compared with Drakkar (Figure 7B). Interestingly, 4 of the most up-regulated genes (c31122, c59522, c26764, c402) in Drakkar code for cysteine proteases known to play an important role in programmed cell death (PCD) associated with the hypersensitive response (HR) [48,49]. Two other highly up-regulated genes (c7149 and c48400) encode germin-like proteins potentially associated with ROS production and cell-wall cross-linking in defense [50]. One up-regulated gene (*Nbi-D - *c37826) has been previously shown to be involved in flax resistance to rust (*Melampsora lini*) [51]. In Belinka, the most highly up-regulated gene (c505) as compared to Drakkar correspond to a hypersensitive-induced response protein.

Overall, these results would suggest that our flax microarray platform is capable of detecting biologically-relevant differential gene expression between contrasted flax varieties. As such, our microarrays represent a powerful tool for identifying candidate genes potentially associated with quality-related polymorphism and therefore represent a valuable contribution to molecular-based plant improvement programs.

## Conclusion

We have developed a powerful and robust high-density oligo-microarray platform for transcriptomics in flax. High correlations were consistently obtained with technical repetitions on a wide range of different samples and results were cross-validated using an independent method (qRT-PCR). The platform is capable of high discrimination and can provide biologically-useful information on specific gene expression profiles of different flax tissues, and developmental stages. Initial studies also enabled the identification of specifically-expressed cell wall- and defence-related genes in 2 different flax varieties showing contrasting fibre quality and resistance towards a fungal pathogen. These results indicate that our microarray platform can make a useful contribution towards understanding the genetic basis of plant quality in flax.

## Methods

### Plant material and tissue collection

*Linum usitatissimum *(Barbara an oil-seed cultivar and Hermes a fibre cultivar)[42] plants were grown in a growth chamber (light/night cycles 16 h (22°C)/8 h (19°C), 50% humidity and light intensity of 400 μE s^-1 ^m^-2^). Individual flowers (Barbara) were tagged at anthesis. The developing seeds of Barbara were collected at 10-15, 20-30 and 40-50 Days After Flowering (DAF), immediately flash-frozen in liquid nitrogen and stored at -80°C until used for experiments. 15 cm-long stem samples were recovered from plants (Hermes) harvested at two different developmental stages: (1) vegetative and (2) green capsule. The outer fibre-bearing tissues were peeled off and inner tissues (xylem) cut into short fragments before both tissues were frozen in liquid nitrogen. Leave samples (Hermes) were collected at vegetative stage, immediately frozen in liquid nitrogen and stored at -80°C. For root samples, plants (Hermes) were grown in a mixture of sand and vermiculites in a growth chamber (light/night cycles 16 h (22°C)/8 h (19°C), 50% humidity and light intensity of 150 μE s^-1 ^m^-2^) for 10 days. Roots were rinsed in cold water (4°C) and the extremity of each root was cut off, frozen in liquid nitrogen, and stored at -80°C.

### RNA extraction

Total RNA was isolated from the collected tissues. 100 mg of frozen seed samples were ground in liquid nitrogen and polyphenols and polysaccharides were precipitated [52] prior to RNA extraction by hot phenol, modified after Verwoerd et al. [53]. 100 mg of frozen stem/root/leaf tissues were ground in liquid nitrogen using the Trizol method (Invitrogen, Carlsbad, CA). Total RNAs were purified using the RNeasy Plant kit (Qiagen) according to the manufacturer's instructions. The genomic DNA was eliminated after treatment with Dnase I for 20 min at 37°C using the DNA-free kit (Ambion, Austin, TX, USA). RNA was checked for purity and degradation by capillary electrophoresis using the Bio-Analyzer Experion (Bio-Rad; RNA Standard Sens kit; RNA StdSens chips). RNA concentration was also determined by spectrometry and only RNAs with an OD260:OD280 ratio of > 1.8 and no discernable degradation were used in PCR-based experiments.

### 454 Sequencing and Bioinformatics

454 GS FLX technology was used to sequence 9 different RNA samples extracted from 2 distinct flax genotypes and different tissues/developmental stages (Table 1). Individual libraries were prepared for each sample using the MID bar-coding system from Roche. Four separate half GS FLX runs were performed (2 total runs) on 4 cDNA library pools: 1) leaf and root; 2) 3 seed developmental stages; 3) Internal/external stem samples, vegetative stage; 4) internal/external stem samples, green capsule stage. Raw data was generated as Sff files. The Sff_extract tool http://bioinf.comav.upv.es/sff_extract/index.html was used to extract reads, qualities and A and B adaptor positions for each sample. A specific search for SMART forward and reverse primers was performed on all reads using cross_match utility.

### Assembly and annotation

Reads from each genotype were identified, and a *de novo *assembly of all reads/samples was performed using the MIRA tool (development version number 2.9.29 × 4). Parameters used were the default ones plus: -job = denovo, est, normal, 454 -GE:not = 8 -GE:kcim = on - LR:mxti = yes -SB:lsd = on -CL:mbc = on. By default, reads smaller than 40 bp are excluded by MIRA. Reads and contigs were then stored in an EST2uni database for data and annotation management.

### Microarray design and oligo synthesis

A total of 384,168 oligonucleotides (25-mers long) were designed and used to construct high-density flax microarrays based on the Nimblegen 385K design format (Nimblegen Systems, Inc., Madison, WI, USA). This design enabled an elevated number (8) of distinct oligos to be used for each of the 48,021 contigs selected from the overall total of 59,000 contigs obtained by assembling the GS FLX sequences. Technical specifications and design files of this new platform for high-throughput analysis of gene expression in flax are publicly available on the GEO website under accession number GPL10419.

### cDNA synthesis, labeling and hybridization

Double-stranded cDNA (ds-cDNA) was synthesized from 10 μg of total RNA using an Invitrogen SuperScript ds-cDNA synthesis kit in the presence of 250 ng random hexamer primers. ds-cDNA was cleaned and labeled in accordance with the Nimblegen Gene Expression Analysis protocol (Nimblegen Systems, Inc., Madison, WI, USA). Briefly, ds-cDNA was incubated with 4 μg RNase A (Promega) at 37°C for 10 min and cleaned using phenol:chloroform:isoamyl alcohol, followed by ice-cold absolute ethanol precipitation. For Cy3 labeling of cDNA, the Nimblegen One-Color DNA labeling kit was used according to the manufacturer's guideline detailed in the Gene Expression Analysis protocol (Nimblegen Systems, Inc., Madison, WI, USA). One μg ds-cDNA was incubated for 10 min at 98°C with 2 OD of Cy3-9mer primer. Then, 100 pmol of deoxynucleoside triphosphates and 100U of the Klenow fragment (New England Biolabs, Ipswich, MA, USA) were added and the mix incubated at 37°C for 2h30. The reaction was stopped by adding 0.1 volume of 0.5 M EDTA, and the labeled ds-cDNA was purified by isopropanol/ethanol precipitation. Microarrays were hybridized at 38°C during 16 to 18 h with 6 μg of Cy3 labelled ds-cDNA in Nimblegen hybridization buffer/hybridization component A in a hybridization chamber (Hybridization System - Nimblegen Systems, Inc., Madison, WI, USA). Following hybridization, washing was performed using the Nimblegen Wash Buffer kit (Nimblegen Systems, Inc., Madison, WI, USA).

### Data Analysis

Slides were scanned at 5 μm/pixel resolution using an Axon GenePix 4000 B scanner (Molecular Devices Corporation, Sunnyvale, CA, USA) piloted by GenePix Pro 6.0 software (Axon). Scanned images (TIFF format) were then imported into NimbleScan software (Nimblegen Systems, Inc., Madison, WI, USA) for grid alignment and expression data analyses. Expression data were normalized through quantile normalization [54] and the Robust Multichip Average (RMA) algorithm [55] included in the NimbleScan software. Identification of genes displaying a change in expression over repetitions was accomplished with a script utilizing library functions in R with a false discovery rate (FDR) of less than 5%. The SAM [56] was used to identify differentially expressed genes over different conditions and log2(ratio) ≥ 1 and ≤ -1 were used for filtering gene expression profiles. Analysis was completed with the PCA module of the J-express program [57]. Functional annotation of differentially-expressed genes was based on Gene Ontology http://www.geneontology.org/. All the microarray data have been submitted to the Gene Expression Omnibus (GEO) database http://www.ncbi.nlm.nih.gov/geo with the accession number GSE21868.

### Quantitative reverse transcriptase-PCR (qRT-PCR) analysis

#### cDNA Synthesis

5 μg aliquots of total RNA were treated with DNaseI using a TURBO DNA*-free *Kit (Ambion) according to the manufacturer's instructions, then first-strand cDNA was synthesized using M-MuLV RNase H^- ^reverse transcriptase (Finnzymes) with 2.5 μg of random hexamers and 500 ng of oligo(dT)12 according to the manufacturer's instructions. The reaction was stopped by incubation at 70°C for 10 min, and the reaction mixture treated with RNaseH (BioLabs) according to the manufacturer's instructions before dilution with 600 μL of sterile de-ionized water.

#### qRT-PCR Experiment Design

Transcript levels were assessed by qRT-PCR, in assays with triplicate reaction mixtures (final volume, 20 μL) containing 5 μL of cDNA, 0.5 μM of both forward and reverse primers, and 1X DyNamo Capillary SYBR Green qRT-PCR mix (Finnzymes). qRT-PCR experiments used a balanced randomized block design, as recently advised [58]. A LightCycler (Roche) was used to acquire the CT values for each sample. The following standard protocol was applied for all amplifications: 10 min at 95°C, followed by 45 cycles of 10 s at 95°C, 15 s at 60°C, and 15 s at 72°C. A melting curve analysis was added to each PCR program and the size of PCR products was assessed by electrophoresis in agarose gels. The primer sequences used for all target genes are presented in Additional file 5.

Relative standard curves describing the PCR efficiencies (E) for each primer pair were generated for each amplicon according to Larionov et al. [59]. Normalization of qRT-PCR was performed using reference genes (R) according to Gutierrez et al. [60]. Twelve genes (Additional file 5) were chosen for their stability across the set of microarray experiments previously shown in this paper. Their expression was assessed by qRT-PCR, and they were ranked according to their stability of expression using geNorm software [61]. The_*c3168 *(lcl|genolin_c3168 628 nt) and *c10916 *(lcl|genolin_c10916 813 nt) genes were the most stably expressed ones among the 12 tested and, thus, were used to normalize the qRT-PCR data. The normalized expression patterns obtained using both reference genes were similar, so only the data normalized with *c10916 (*highly similar to a Dehydrodolichyl diphosphate synthase 6 of *Arabidopsis thaliana*) are shown in this article. Gene expression was calculated using CT and E values with the formula, E_T_^(CT^_tissueA_^-CT^_tissueB_^)^/E_R_(^(CT^_tissueA_^-CT^_tissueB_^)^, where (T) is the target gene and (R) the reference gene, (tissueA) is related to cDNA from the tissue showing the lowest expression and (tissueB) from the tissue showing the highest expression. All qRT-PCR results represent means as calculated from the three technical replicates [58].

## Authors' contributions

BT conceived and designed the project. BT and RT grew flax plants and provided samples. BT and BC characterized the biological materials. NR and JD managed the 454 sequencing, data assembly, conceived and constructed the database and oligo design. AL, SF and OW performed microarrays experiments. LG validated array data with qRT-PCR. SH managed manuscript preparation. All listed authors edited the manuscript. All authors read and approved the final manuscript.

## Supplementary Material

Additional file 1 **Analyses of differential gene expression between stem inner tissues and stem outer tissues sampled at vegetative and green capsule stages**. Lists of differentially expressed genes and GO analyses (biological processes) for i) stem inner vegetative (SIV) vs stem outer vegetative (SOV), ii) stem inner green capsule (SIGC) vs stem outer green capsule (SOGC), iii) stem inner vegetative (SIV) vs stem inner green capsule (SIGC), iv) stem outer vegetative (SOV) vs stem outer green capsule (SOGC). Click here for file

Additional file 2 **Analyses of differential gene expression (selected functional groups) between stem inner tissues and stem outer tissues sampled at vegetative and green capsule stages**. Lists of differentially expressed genes (selected functional groups only) for i) stem inner vegetative (SIV) vs stem outer vegetative (SOV), ii) stem inner green capsule (SIGC) vs stem outer green capsule (SOGC), iii) stem inner vegetative (SIV) vs stem inner green capsule (SIGC), iv) stem outer vegetative (SOV) vs stem outer green capsule (SOGC). Lists of differentially expressed genes and GO analyses (biological processes) for i) stem inner vegetative (SIV) vs stem outer vegetative (SOV), ii) stem inner green capsule (SIGC) vs stem outer green capsule (SOGC), iii) stem inner vegetative (SIV) vs stem inner green capsule (SIGC), iv) stem outer vegetative (SOV) vs stem outer green capsule (SOGC). Click here for file

Additional file 3 **Differentially expressed genes between Drakkar and Belinka flax cultivars**. List of differentially expressed genes between Drakkar and Belinka flax cultivars. Values for up-regulated genes are represented in red and those for down-regulated genes in green colour. Click here for file

Additional file 4 **Number of differentially expressed genes between Drakkar and Belinka flax cultivars represented as GO biological process**. Drakkar specifically expressed genes are represented as dark columns and those of Belinka as clear columns. Click here for file

Additional file 5 **Primer sequences used in qRT-PCR analyses**. Sequences of primers used for quantifying target genes by qRT-PCR (Table 1) and Sequences of primers for putative reference genes tested by geNorm (Table 2). Click here for file
