# Supplementary material for: Development and validation of a flax (Linum usitatissimum L.) gene expression oligo microarray
Source: BMC Genomics. 2010 Oct 21;11:592. doi: 10.1186/1471-2164-11-592 (PMC3091737; doi:10.1186/1471-2164-11-592)
Supplement: Additional file 4 — Number of differentially expressed genes between Drakkar and Belinka flax cultivars represented as GO biological process. Drakkar specifically expressed genes are represented as dark columns and those of Belinka as clear columns. [file 1471-2164-11-592-S4.DOC]

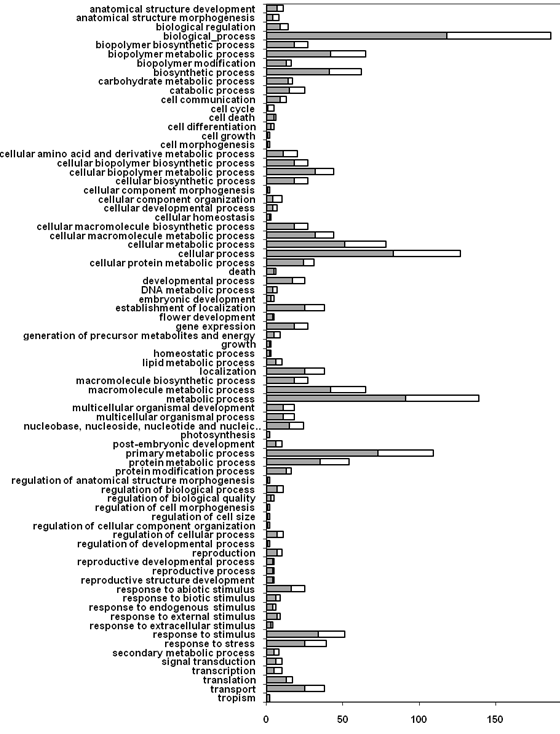


**Supplementary data file 3**. Number of differentially expressed genes between Drakkar and Belinka flax cultivars represented as GO biological process. Drakkar specifically expressed genes are represented as dark columns and those of Belinka as clear columns.
