# Supplementary material for: Development and validation of a flax (Linum usitatissimum L.) gene expression oligo microarray
Source: BMC Genomics. 2010 Oct 21;11:592. doi: 10.1186/1471-2164-11-592 (PMC3091737; doi:10.1186/1471-2164-11-592)
Supplement: Additional file 5 — Primer sequences used in qRT-PCR analyses. Sequences of primers used for quantifying target genes by qRT-PCR (Table 1) and Sequences of primers for putative reference genes tested by geNorm (Table 2). [file 1471-2164-11-592-S5.DOC]

| **Table 1: Sequences of primers used for quantifying target genes by qRT-PCR** | | | | |  |
| --- | --- | --- | --- | --- | --- |
| **Amplicon name** | | **Forward Primer** | | **Reverse Primer** | |
| c20715 | | CCCTTTTGCAAATGATGGTC | | CATCAGTCTCGACGAGGTCA | |
| c2491 | | GTTGTCCCGAAACGGAGTTA | | CGTGGACCAAGCTAGGGTTA | |
| c602 | | GGCCTCCGACATAATCAAGA | | CGTTGACCATCTGGTTGTTG | |
| c21991 | | TTCCGGTGGATTTCAGTGAT | | TTTCGCGATACCTTTCCTTG | |
| c24118 | | TATGCGTTCATTGGGACAGA | | AGGAACTCCGGGAAATCAAT | |
| c9380 | | TGCTGGACTGTTCAATTTCG | | ACGGCAATGAATGACTGTGA | |
| c2533 | | GGGAGTTGTTGCAGGTGTTT | | ACGGGTAGAGATGGACGATG | |
| c3323 | | CAAACCACAAACGCCCTACT | | CGGAGAGAAGGGAGGGATAC | |
| c4370 | | TATCCGACGACGCTAAATCC | | CCTCGGTAGCAATTGGGTTA | |
| **Table 2: Sequences of primers for putative reference genes tested by geNorm** | | | | |  |
| **Amplicon name** | **Forward Primer** | | **Reverse Primer** | |  |
| s_c3168 | GACTCGTTCCTGAGGTCTGC | | CCATCACACCCACAGTTCAG | |  |
| s_c7348 | TGGTATTCTGCCTTCCTTGG | | CCAGGACTTAGCTCGTCCAC | |  |
| c1016 | ACATGGCCATTGTGCATAGA | | TGGTCTCGAAAGCATCTTCA | |  |
| c10026 | GGGCTGCACATATCGAGTTT | | CGATTGTCGAGTGAAAACGA | |  |
| c10679 | AACAGCTCGCCAGGGTACTA | | ATCCGATGATACGGACGAAG | |  |
| c10916 | GTGGCTCCTGATCCAGACAT | | CAACATGCCACAGACCAATC | |  |
| c11712 | CTCTCTCGGAACTGGAGGTG | | GCGATTTCAGCAACAACTCA | |  |
| c11721 | CAAGGAACGAGCTGAAAACC | | TTCACGGAATCCGACTTTTC | |  |
| c12095 | CTGGTCAGCTGCATCAGGTA | | TGTTTCTGGTTCGTCATCCA | |  |
| c12179 | GGCTTCAGCAGAAAGTTTGG | | ACCTCTTTGGCATCCTTCCT | |  |
| c13139 | ATTACGGACGGACTTCGAGA | | GGTGGTGTTCTTGGACGAGT | |  |
| c13148 | GGCGAAGGAGAAAAGTGATG | | TTCCTCCTTTCCATGACACC | |  |
